# Supplementary material for: Modelling the effectiveness of antiviral treatment strategies to prevent household transmission of acute respiratory viruses
Source: PLoS Comput Biol. 2024 Dec 5;20(12):e1012573. doi: 10.1371/journal.pcbi.1012573 (PMC11620401; doi:10.1371/journal.pcbi.1012573)
Supplement: S1 Text — (PDF) [file pcbi.1012573.s001.pdf]

Supporting information S1 Text: Modelling the effectiveness of antiviral treatment strategies to prevent household transmission of acute respiratory viruses

Hind Zaaraoui, Clarisse Schumer, Xavier Duval, Bruno Hoen, Lulla Opatowski, Jérémie Guedj

Viral load dynamic model and calibration

Data

We analyzed SARS-CoV-2 viral load data collected during untreated infection in the National Basketball Association's cohort, using data already analyzed and publicly available [1]. The dataset contains 1510 infections in 1443 individuals infected with Alpha, Delta and Omicron variants.
For the sake of our analysis, we focused on primary infections and in individuals in which the delay between the last negative PCR test and the first PCR test did not exceed 4 days. The latter cutoff allowed to focus on individuals for which the time window for infection was not too large [2]. The final dataset contained 605 infections, with 62% caused by omicron, 9% by delta, 23% by other lineages and 6% by unreported lineages (see Fig A).

Model selection

To develop the viral dynamic model, we used a step-wise approach, selecting the model based on the lowest Bayesian Information Criterion corrected (BICc) value. Our baseline model was a model with an innate immune response modelled through a refractory state induced by interferons (IFNs) and an adaptive immune response modelled through a changing cell death rate. We then evaluated whether incorporating a secondary IFN effect on key viral parameters such as viral transmission rate, production rate, clearance rate and cell death rate, improved model performance. The equations are provided in Fig B.
The parameters of the models were estimated by computing the maximum likelihood estimator using the stochastic approximation expectation-maximization (SAEM) algorithm implemented in Monolix Software 2023R1.

Assumption on parameter values and initial condition

To ensure parameter identifiability, several parameters were fixed. The virion clearance rate c, was set to 10 d^{-1} similar to previous findings [3]. The proportion of infectious viruses was assumed to be 10^{-4} of the total RNA viral load [4]. The duration of the eclipse phase was set to 0.25 d. The rate at which refractory cells come back to susceptible cells was fixed to 0.34 d^{-1} [5].

In each individual, we estimated the time of infection t_{inf} using the same method as in [3]. We have bounded t_{inf} by the maximal duration between detection and infection (4days.) Then, for $t \leq t_{inf}$, the initial conditions are $T = 1.33 \times 10^5$,$R = 0$,$I_1 = 0$,$I_2 = 1/30$,$V_I = 0$,$V_{NI} = 0$,$F = 0$. In our work, we translated the infection time to $t_{inf} = 0$.

Selection of model

The selected model based on the BICc contains a reduction in viral production mediated by IFNs. The BICs for all tested models are presented in the following table. The final model incorporates an innate immune response through a refractory state

**Models**	**BICc**
Base Model	19912.47
M_1	19592.48
M_2	18944.51
M_3	19009.41
M_4	19114.96

Table 1. BICc of models tested

and a reduction in viral production, both induced by IFNs, along with an adaptive immune response represented by a variable cell death rate. The parameters of the model are given in the S1 Table.
A visual predictive check (VPC) shows that the model's predictions align well with the observed data, as shown Fig C.

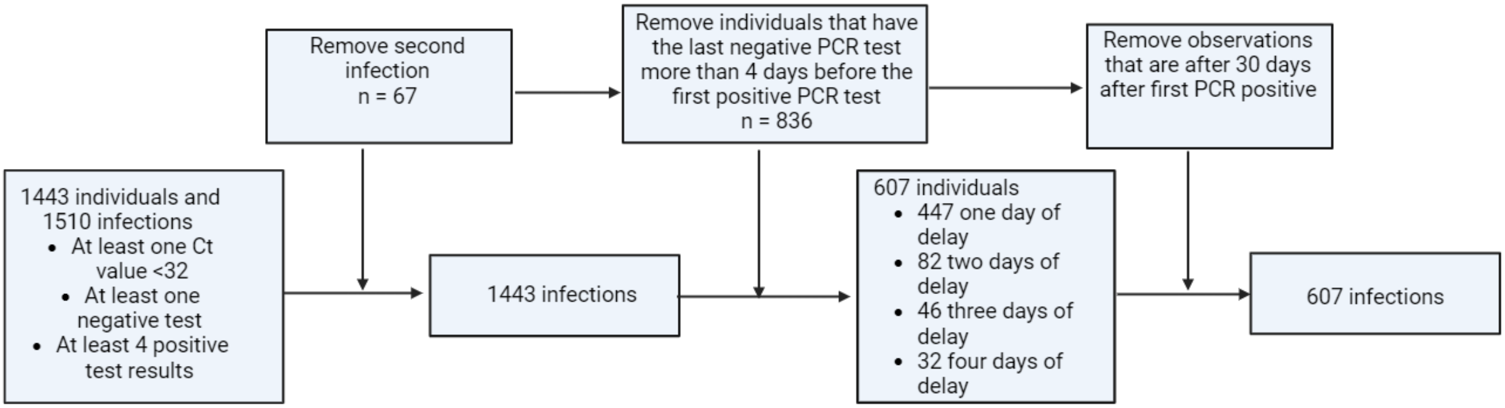

Fig A. Flowchart of SARS-CoV-2 data refinement

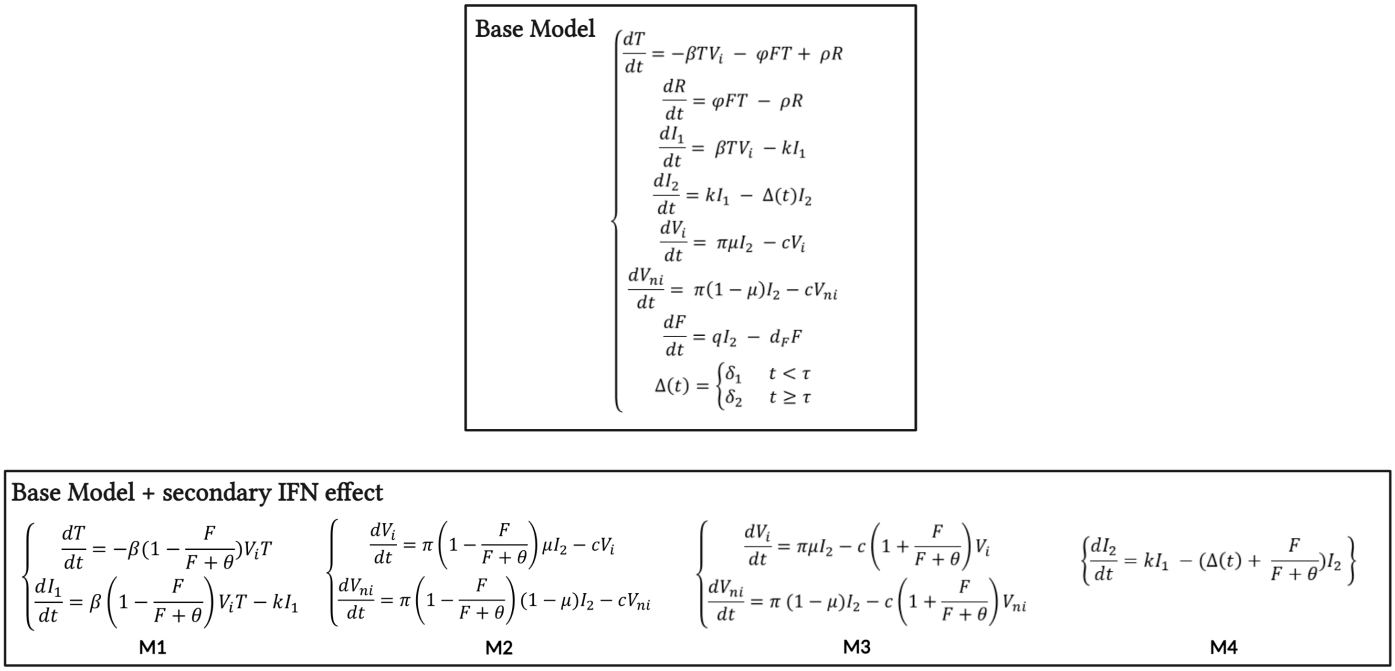

Fig B. Selection of models

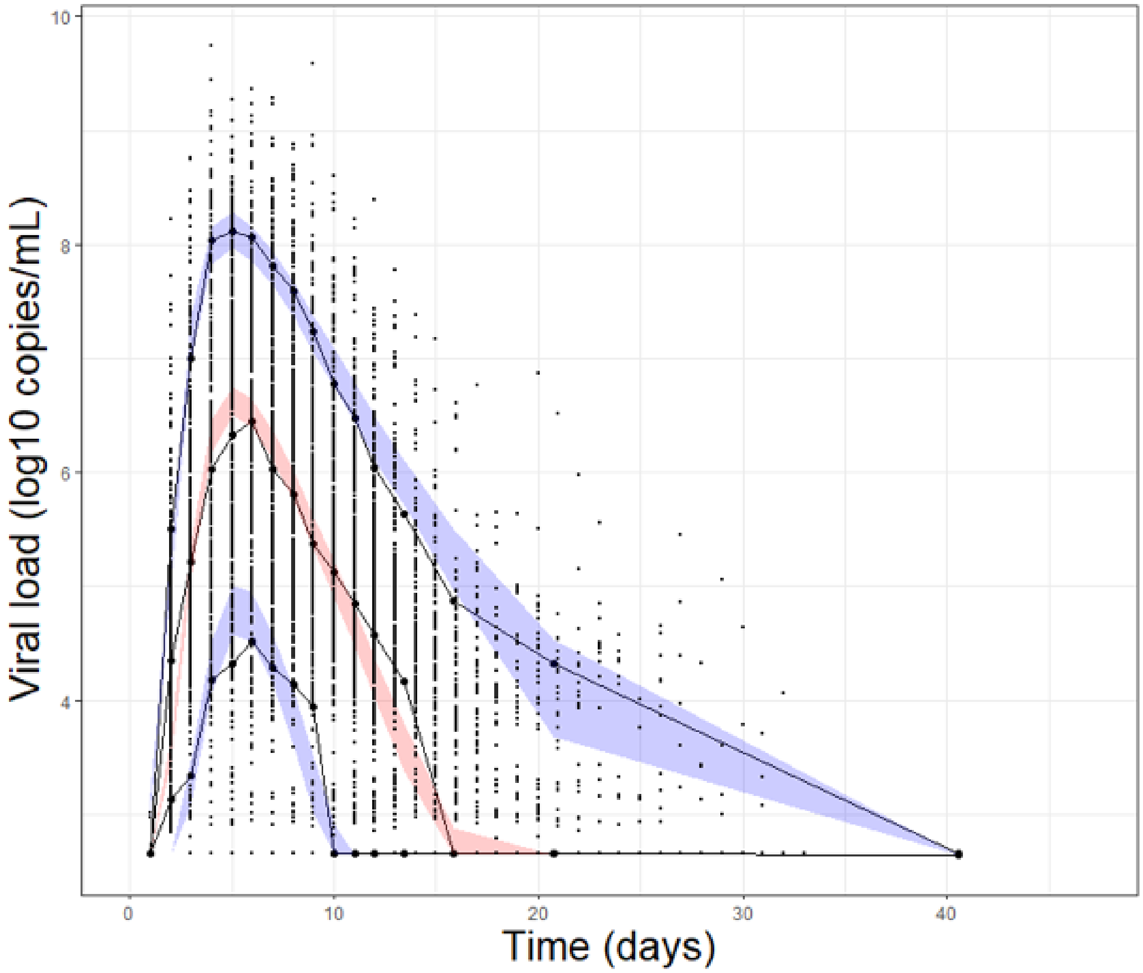

Fig C. Visual predictive check of final model

References

1. Owens K, Esmaeili-Wellman S, Schiffer JT. Heterogeneous SARS-CoV-2 kinetics due to variable timing and intensity of immune responses. JCI Insight, 2024;9(9):e176286.

2. Killingley B, Mann AJ, Kalinova M, Boyers Al, Goonawardane N et al. Safety, tolerability and viral kinetics during SARS-CoV-2 human challenge in young adults. Nature Medicine, 2022, 28(5), 1031-1041.

3. Néant N, Lingas G, Le Hingrat Q, Ghosn J, Engelmann I, Lepiller Q et al. Modeling SARS-CoV-2 viral kinetics and association with mortality in hospitalized patients from the French COVID cohort. Proceedings of the National Academy of Sciences, 2020, 118(8), e2017962118.

4. Gonçalves A, Bertrand J, Ke R, Comets E, De Lamballerie X, Malvy D et al. Timing of antiviral treatment initiation is critical to reduce SARS-CoV-2 viral load. CPT: pharmacometrics and systems pharmacology, 2020, 9(9), 509-514.

5. Pizzorno A et al. Interactions Between Severe Acute Respiratory Syndrome Coronavirus 2 Replication and Major Respiratory Viruses in Human Nasal Epithelium. The Journal of infectious diseases vol. 226,12, 2022, 2095-2104.
