# Supplementary material for: Modelling the effectiveness of antiviral treatment strategies to prevent household transmission of acute respiratory viruses
Source: PLoS Comput Biol. 2024 Dec 5;20(12):e1012573. doi: 10.1371/journal.pcbi.1012573 (PMC11620401; doi:10.1371/journal.pcbi.1012573)
Supplement: S2 Fig — (A,C,E,G) Number of infected individuals per 1000 households. (B,D,F,H) Virological burden. Gray: I0; orange dashed line: Icur; orange line: Icur + Hpep; green dashed line: Ipep; green line: Ipep + Hprep. Results are shown by household sizes, S = 2 (darkest color) to S = 6 (lightest color). (PDF) [file pcbi.1012573.s003.pdf]

S2 Figure: Modelling the effectiveness of antiviral treatment strategies to prevent household transmission of acute respiratory viruses

Hind Zaaraoui, Clarisse Schumer, Xavier Duval, Bruno Hoen, Lulla Opatowski, Jérémie Guedj

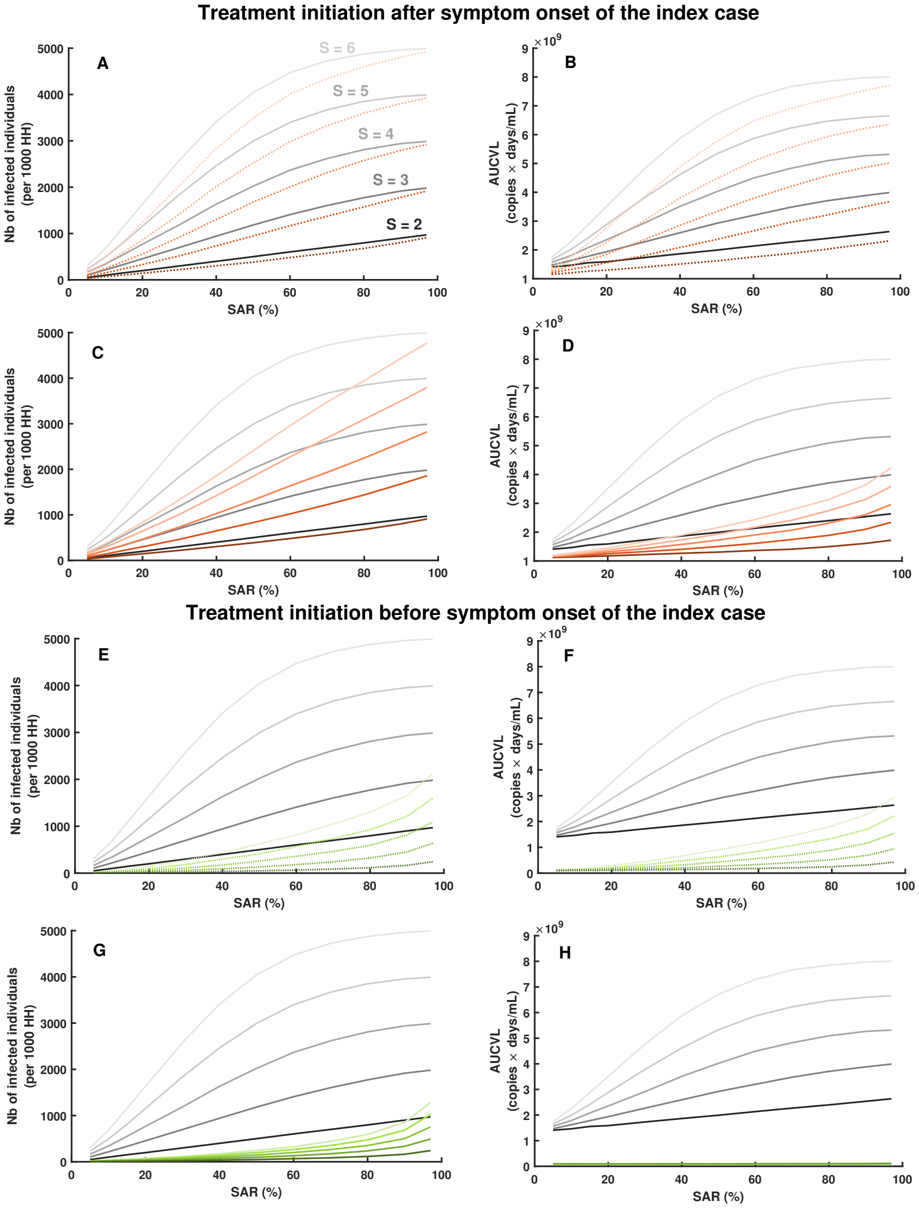

S2 Fig. Transmission and virological burden according to household size for different treatment timings. (**A,C,E,G**) Number of infected individuals per 1000 households. (**B,D,F,H**) Virological burden. Gray: I_0; orange dashed line: I_{cur} ; orange line: $I_{cur} + H_{pep}$; green dashed line: I_{pep}; green line: $I_{pep} + H_{prep}$. Results are shown by household sizes, S=2 (darkest color) to S=6 (lightest color).
