# Supplementary material for: Modelling the effectiveness of antiviral treatment strategies to prevent household transmission of acute respiratory viruses
Source: PLoS Comput Biol. 2024 Dec 5;20(12):e1012573. doi: 10.1371/journal.pcbi.1012573 (PMC11620401; doi:10.1371/journal.pcbi.1012573)
Supplement: S1 Table — (PDF) [file pcbi.1012573.s013.pdf]

S1 Table: Modelling the effectiveness of antiviral treatment strategies within household transmission of acute respiratory viruses

Hind Zaaraoui, Clarisse Schumer, Xavier Duval, Bruno Hoen, Lulla Opatowski, Jérémie Guedj

References

1. Ke R, Zitzmann C, Ho DD, Ribeiro RM and Perelson AS. In vivo kinetics of SARS-CoV-2 infection and its relationship with a person's infectiousness. Proceedings of the National Academy of Sciences, 2021, 118(49), e2111477118.

2. Marc A, Kerioui M, Blanquart F, Bertrand J, Mitjà O, Corbacho-Monné M et al. Quantifying the relationship between SARS-CoV-2 viral load and infectiousness. Elife, 2021, 10, e69302.

**Within-host parameters**	**Description**	**Fixed effect (RSE %)**	**Random effect SD* (RSE %)**
β	Infection rate	$1.89 \times 10^{-5}(12.3)$	0.699(3.17)
τ	Adaptive response time	14.47(0.704)	0.401(4.13)
δ_1 (d^{-1})	Loss rate of infected cells at $t < \tau$	0.976(0.523)	0.231(6.64)
δ_2 (d^{-1})	Loss rate of infected cells at $t \geq \tau$	3.56(7.95)	0.27(5.64)
π $(cp \times mL^{-1}d^{-1})$	Rate of viral production	$6.43 \times 10^5(12)$	-
c (d^{-1})	Virion clearance rate	10	-
k (d^{-1})	Rate of transition to productively infected cells	4	-
μ	Proportion of infectious virus	0.0001	- -
ϕ (d^{-1})	Refractory rate of target cells	0.00124(19)	0.266(122)
d_F (d^{-1})	Loss rate of effectors	3.03(10.7)	0.83(5.26)
θ	Half-maximal concentration of effectors	1720(18.3)	1.55(4.85)
ρ (d^{-1})	Rate of transition from refractory to susceptible cells	0.00431(10.4)	0.123(103)
ϵ	Treatment efficacy	99%	0.380

**Between-host parameters**	**Description**	**Parameter value**	**SD of the random effects***
M $(cp^{-1} \times mL)$	Viral load-transmission association	$[2.9 \times 10^{-7} - 7.9 \times 10^{-5}]$	0.85
h	Steepness of the viral load-transmission association	0.49	-

S1 Table. Parameter values used to generate viral dynamic profiles and transmission probabilities [1, 2]. * All parameters are assumed to have a log-normal distribution for the random effects, except ϵ, with a logit distribution. Parameters with no random effects (same parameter value in all individuals) are indicated with "-". *cp = copies*
