# Supplementary material for: Modelling the effectiveness of antiviral treatment strategies to prevent household transmission of acute respiratory viruses
Source: PLoS Comput Biol. 2024 Dec 5;20(12):e1012573. doi: 10.1371/journal.pcbi.1012573 (PMC11620401; doi:10.1371/journal.pcbi.1012573)
Supplement: S2 Table — (PDF) [file pcbi.1012573.s014.pdf]

S2 Table: Modelling the effectiveness of antiviral treatment strategies to prevent household transmission of acute respiratory viruses

Hind Zaaraoui, Clarisse Schumer, Xavier Duval, Bruno Hoen, Lulla Opatowski, Jérémie Guedj

Household size	Proportion
2	50.9%
3	21.6%
4	18.1%
5	6.7%
6	2.6%

S2 Table. Proportion of household sizes in France [1].

References

1. INSEE. https://www.insee.fr/fr/statistiques/2381486tableau-figure1-radio1. 2016
