# Supplementary material for: Modelling the effectiveness of antiviral treatment strategies to prevent household transmission of acute respiratory viruses
Source: PLoS Comput Biol. 2024 Dec 5;20(12):e1012573. doi: 10.1371/journal.pcbi.1012573 (PMC11620401; doi:10.1371/journal.pcbi.1012573)
Supplement: S3 Table — (PDF) [file pcbi.1012573.s015.pdf]

# S3 Table: Modelling the effectiveness of antiviral treatment strategies to prevent household transmission of acute respiratory viruses

Hind Zaaraoui, Clarisse Schumer, Xavier Duval, Bruno Hoen, Lulla Opatowski, Jérémie Guedj

| Time to peak VL | Within-host parameters | Value | SD |
|---|---|---|---|
| **1d** | $\beta_{1d}$ | $\beta_{4d} + 60 \times 10^{-5}$ | $SD_{4d} + 1.6$ |
| **7d** | $\beta_{7d}$ | $\frac{\beta_{4d}}{3}$ | $\frac{SD_{4d}}{3.5}$ |

**S3 Table. Modified parameters in the viral load model (see Eq. 1 of the main paper) to reproduce time to peak viral load equal to 1 and 7 days.** Parameters used to reproduce a time to peak viral load ($\beta$) are given in S1 Table.
