# Supplementary material for: Modelling the effectiveness of antiviral treatment strategies to prevent household transmission of acute respiratory viruses
Source: PLoS Comput Biol. 2024 Dec 5;20(12):e1012573. doi: 10.1371/journal.pcbi.1012573 (PMC11620401; doi:10.1371/journal.pcbi.1012573)
Supplement: S1 Code — The code is protected by Agence de Protection des Programmes (APP) in France, and we have the permission to share it. (ZIP) [file pcbi.1012573.s016.zip › Code and code description/README file - Program description- PLOS COMP BIO.pdf]

README file

All the simulations of the paper “Modelling the effectiveness of antiviral treatment strategies to prevent household transmission of acute respiratory viruses”, of the Manuscript ID: PCOMPBIOL-D-23-01419R1, are done in MATLAB R2022a.

The link to install MATLAB: https://fr.mathworks.com/help/compiler/install-the-matlab-runtime.html?searchHighlight=installer%20R2022a&s_tid=srchtitle_installer%20R2022a_2

All the data used has been simulated and integrated in the file in .CSV Excel file format.

The **Code** file includes all the program functions and some data .csv files simulated of the viral load dynamics and of some outcomes of the impact of different treatment strategies on transmission and viral burden. The MATLAB functions provided computes:

1.The intra-hosts simulation model: viral load dynamics simulation within individuals

2.The inter-hosts simulation model: transmission simulation within households

1. Viral load simulation

a. The code

The code of the viral load simulation is defined in **“`viral_loads`”** function where the input are “`(treat_initiation,epsilon_treat,N,peaktime)`” where `treat_initiation` is the treatment time initiation from -5 (for treatment before infection) to 7 (12 days after infection). N is the number of individuals that we compute for each one their viral load (VL) dynamics. `epsilon_treat` is the treatment efficacy (99% in our case or can take any value), and `peaktime` is the average peak time of the viral load. The program computes the VL dynamic using parameters of Table 1 of the VL. The differential equation system describing the evolution the number of target cells and the viral load (V) is defined by the function “drug_effect_immu”. This function has to be run firstly before **“`house_simulation`”** function.

- The run time of this function for 100,000 individuals is about 10 minutes

b. The code output

The output is Excel file .csv of viral load dynamics for N individuals during time includes only 2 values of viral load per day for 25 days since infection. The values of viral load are either without treatment (column 3) or with treatment (column 4). The treatment is initiated from infection time to 13 days after infection. Sometime an individual may be treated even if he is not infected, as prophylaxis, and the data are represented in “`ViralLoad-5_dos0.99`” file if the treatment is of 99% efficacy.

For example, in the Excel file "`ViralLoad-4_dos0.99`" we have the viral loads without and with treatment of efficacy `99%` initiated 1 day after infection (i.e. `4 days before` the symptoms on average). For "`ViralLoad2_dos0.99`" the treatment is initiated `2 days after` time symptom onset on average which is 7 days after infection with treatment efficacy of `99%=0.99.` The treatment efficacy can be modified to any epsilon (50% and 90% were our examples in our paper).

2. Household transmission simulation

a. The code

The code of the household viral transmission simulation is defined in **"house_simulation"** function where the input are (`N, incubation, epsilon_treat, strat_treat, time_treat_index_sympt, peaktime`) where N is the number of households (HH) of the simulation (in our work we took 50,000 HH). `Incubation` is the incubation period that may be on average either 5 days or 3 days. `epsilon_treat` is the treatment efficacy, `strat_treat` is the treatment strategy that can take as value "no treatment", "only index" or "all HH", and `strat_treat` is initiating treatment either for post-symptomatic index with input "after symptoms" or pre-symptomatic index with input "before symptoms".
The code takes the information of the viral load dynamics of individuals, saved in the .CSV files, that will be used for the indexes and for the infected individuals within HH. The AUCVL (see the paper) is computed in this code by summing the "`AUCLog_without_protocole1`", vector of the AUC of infected individuals without treatment, "`AUCLog_treat_protocole1`", vector of the AUC of infected individuals with treatment.
This program computes also the instantaneous transmission probability thanks to the power model defined in the paper with "**proba_transm_pow**" function taking into account the VL dynamics for the infected individuals. When an individual is infected, it has a probability "XX_i" to infect another individual. Hence we simulate a Bernoulli variable of parameter "XX" vector of "XX_i" thanks to "`TABLE_transmission=(UU<= XX)`" where UU is Uniform random variable in interval [0,1], and XX is the vector of instantaneous transmission probability of all the individuals infected at this time.

b. The code output

The **"house_simulation"** function save the results for N households of each size K (from 2 to 6 individuals) and for each different mean direct attack rate at then end of outbreak which is the SAR (from 5% to 97% -12 values of SAR-, see Methods in the Paper). This function gives all the transmission and viral burden outcomes

defined in the paper: such as the number of infected individuals and final attack rates for transmission and AUCVL for the household's viral burden outcome.
For example, one of the saved outcome file:
"`AUC_perHH_treated_simulated0.90_all HH_incub5_before symptoms`" is a file of the AUCVL of each household for all the household sizes, where `ALL` the household is treated with efficacy of treatment of `90%` when the index is identified as a contact case (pre-symptomatic defined in the "`before symptoms`") with incubation period of 5 days (`incub5`).
"`AUC_perHH_treated_simulated0.90_only index_incub5_before symptoms`" is the same results but now if `Only the index` is treated.
Other outputs are also considered as the AUCVL averaged in "`AUCmean_simulated…`", number of non-infected individuals in "`casEvite_simulated…`" files, the final attack rate (FAR) per each HH in "`FAR_perHH_simulated…`" files…

- The run time of this function for 50,000 Households for one treatment efficacy and for 12 different SAR is more than 3 hours approximately per one type of virus (one type of incubation period and one type of averaged viral peak time – from 1 to 7 days after infection -).

3. Resulted plots of the paper

All the curves, bar plots, and tables are computed by these 2 following MATLAB functions: "**Treatment_impact**" and "**Effect_treatment_tables**".

In the "**Treatment_impact**" function, we used all the saved outcomes data of households of different size from the **"`house_simulation`"** function to compute the effectiveness on the number of individuals infected, on the AUCVL with the same inputs and the cost effectiveness of treatment (computation of number of individuals avoided per number of treatments).
This function provides also different treatment impact results such as the number of cases avoided per number of allocated treatments, treatment effectiveness on transmission and viral burden for different treatment strategies curves for different SAR not modified and modified (see section Methods of the paper) …

In the "**Effect_treatment_tables**" function, we provide the final results, in tables format, of treatment effectiveness on transmission and on viral burden for different virus cases with different time peaks (1-, 3-, 5- and 7-days mean peak time after infection if the parameter of the viral load differential equation in **"`viral_loads`"** are `modified as described in our paper`).

- The run time of these functions is about few seconds to few minutes.
