# Supplementary material for: Modelling the effectiveness of antiviral treatment strategies to prevent household transmission of acute respiratory viruses
Source: PLoS Comput Biol. 2024 Dec 5;20(12):e1012573. doi: 10.1371/journal.pcbi.1012573 (PMC11620401; doi:10.1371/journal.pcbi.1012573)
Supplement: Attachment — Submitted filename: Response to reviewers.pdf [file pcbi.1012573.s017.pdf]

Modelling the effectiveness of antiviral treatment strategies to prevent household transmission of acute respiratory viruses

Hind Zaaraoui, Xavier Duval, Bruno Hoen, Lulla Opatowski, Jérémie Guedj

Responses to referees and editor

We thank the reviewers and the reviewing editor for their insightful comments.

Please find our answers below each comment.

Editor

1. **Table S1, please provide the references for all parameters which were not estimated from data**

We have now added in Table S1 the value and reference for all parameters used, which ensures a full reproducibility of our results on both within- and between-host components, and we describe them below.

More broadly, the reviewers have commented upon the fact that the model parameters would be calibrated on SARS-CoV-2 pre-Omicron virus. In this revised manuscript we have clarified that the purpose of our model is **not to focus on a specific variant or a specific virus**. Rather, we propose a model that is sufficiently flexible to reproduce the main patterns of viral dynamics observed for different acute respiratory viruses. If our main analysis is indeed inspired by the viral dynamics of SARS-CoV-2 pre-Omicron variants (with a peak viral load that coincides with symptom onset and occurs 5 days after infection), we also evaluate the results obtained with different time to peak (1-, 3-, 5- and 7-days post infection), and a shorter incubation period of 3 days. This allows us to present in the final figure of the manuscript a general analysis that can apply to different respiratory viruses (SARS-CoV-2 Omicron, IAV, RSV, HRV). Further, and this is also an essential aspect of our analysis, our analysis is not restricted to one specific value for the secondary attack rate (SAR), but rather **evaluates a large range of SAR values between 5 and 97%, therefore exploring all potential levels of contagiousness**.

Another important remark from the reviewers was to ensure reproducibility of model simulations. Viral kinetic parameters have been previously estimated on more than 4,000 SARS-CoV-2 infected individuals ([1], full paper awaiting approval from Regeneron for submission), with data originating from two clinical studies (NCT04425629 and NCT04666441) evaluating the efficacy of the

monoclonal antibody combination REGN-COV2 against SARS-CoV-2. Parameters are summarized in S1 Table and reproduce the typical pattern of viral kinetics of SARS-CoV-2 pre-Omicron virus, with a peak viral load at day 5 post infection and a median time to viral clearance of 20 days, i.e., 15 days post symptom onset (see [10] for a meta-analysis of viral kinetic profiles).

For the between-host component, the model relating the probability of transmission, p(t), during a contact between an uninfected individual and an infected individual with viral load V(t) was proposed by Ke et al [2]:

$$p(t) = 1 - e^{-MV(t)^h}$$

In order to account for the fact that the transmission is not simply proportional to viral load level but depends on many other factors, we generalized this model by assuming that M is log-normally distributed with a mean m and a standard deviation fixed to 0.49, as estimated in [3]. We found that m varying from 4×10^{-7} to 1×10^{-4}mL/copies mimic SAR ranging from 5 to 97%.

In order to clarify all these hypotheses, and following the comment from reviewer 1, we have now added in Table 1 a description of the main model hypotheses. We also rewrote all sections alluding to model parameters (see section “Model calibration”, “Secondary Attack Rate and household size”, “Intervention strategies and measure of their effectiveness” and “Evaluating the impact of different viral kinetic patterns” in Method section).

2. The main study finding is sensitive to viral dynamics and epidemiological characteristics of SARS-CoV-2, which has been changing between variants. For example, the peak viral load for omicron variant appeared several days after symptom onset (PMID: 37768707). For relevance, the authors should consider using parameters from more recent variants.

While the Editor is correct that the central scenario of our analysis relies on typical within host parameters of SARS-CoV-2 pre-Omicron virus, we show that our results are not sensitive to the studied virus for the following reasons:

- As detailed above, our analysis does not assume a fixed transmission rate. While SARS-CoV-2 pre-Omicron virus is typically associated with a SAR~40% (see section “Model calibration” in Methods), our analysis provides the outcome of interventions for SAR ranging from 5 to 97%, i.e., considering all possible outcomes occurring with a poorly or highly transmissible virus.
- We generalize our approach (Figure 7) to provide an exhaustive analysis considering different within-host patterns covering a larger spectrum of acute respiratory viruses, where the peak of viral load can occur either 1, 3 or 7 days, and the incubation period is 3 days (as indeed observed for SARS-CoV-2 Omicron virus).

Review 1

While the study is theoretically interesting and holds potential for practical application in public health and clinical science, it heavily relies on viral load data alone. To strengthen its validity, I recommend validating the proposed computational approach using household epidemiological or clinical data. Although I recognize the value of this study as a simulation work, interpreting its findings for public health practice may present challenges or even pose risks. Regrettably, I am unable to endorse the publication of this paper in Nature Communications.

We thank you for this important comment. As you point out, a key feature of this multi-scale approach is to characterize the association between viral dynamics and transmission. Validating this model on longitudinal data would require designing specific epidemiological studies, involving prospective samplings in household members, along with detailed information on contact patterns. To the best of our knowledge, such study has not been performed. It is precisely one of the goals of our study to support such an investigation in the future.

While it is therefore not possible to address your comment with fine grain information combining simultaneously both scales, we now provide resulting epidemiological outcomes from the model and show their consistency with reported characteristics of SARS-CoV-2 outbreaks. We believe these new elements contribute to better understanding how the two scales are connected and validate its usage to explore scenarios.

Indeed, while our analysis covers all spectrum of SAR values (see below), we also focus on two specific values corresponding to SAR = 40% and 80%, which are the values that have been reported for pre-Omicron variants ([20]-[22]) and Omicron values [23]. For these specific values of SAR, we now provide the generation time predicted by the model (see Fig 2E and F), with a mean value of 5.1 and 4.5 days, respectively. This is very close to the values reported in the literature (initially 7 days and decreased later to 4-5 days [24] and [25]), including in household studies (3 to 7 days in [6], [7] and [9]). Although this does not validate our model *per se*, it shows that it can reproduce patterns of infection dynamics that are consistent with what has been observed in epidemiological studies.

This aspect is now discussed in "Model calibration" as follows: *"The within- and between- host model can generate usual metrics of epidemiological studies, such as the SAR or the generation time, defined as the time interval between the infection of an individual and the infection time of its infector. For instance,* $m = 4.1 \times 10^{-6}$ *mL/copies corresponds to SAR equal to 40%, as typically observed with SARS-CoV-2 pre-Omicron variants [29], leading to a mean generation interval of 5 days, as reported in the literature [46-49]. Similar calculations can be done to reproduce values observed with SARS-CoV-2 Omicron variants (Fig 2)"*

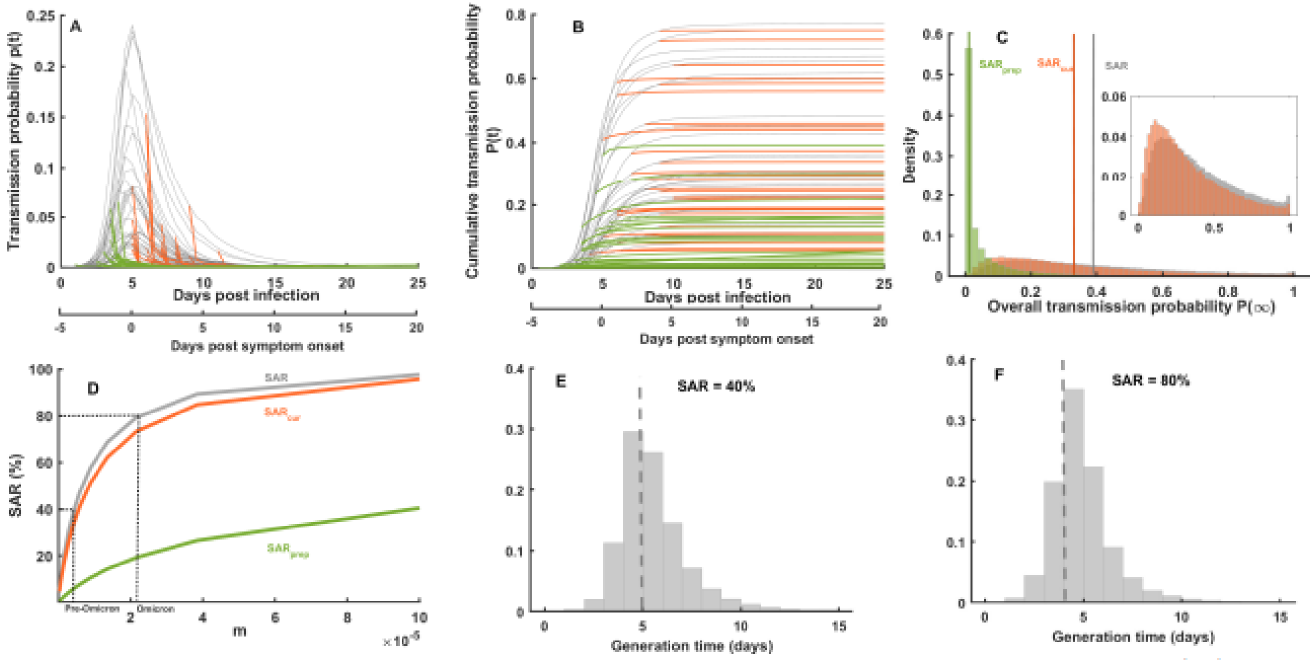

Fig 2. Impact of antiviral treatment on the risk of virus transmission. Impact of antiviral treatment on the risk of virus transmission. **(A)** Instantaneous probability of virus transmission, p(t), in 30 individuals that are either left untreated (I_0, gray), treated within 5 days from symptom onset (I_{cur}, orange), or treated within 5 days after infection (I_{pep}, green). All parameters are given in S1.Table. **(B)** Cumulative transmission probability to another member of the household, P(t), assuming two contacts per day. **(C)** Distribution of the overall transmission probability, $P(\infty)$ without treatment, with SAR defined as the mean of the distribution. Similar definitions are used to defined SAR when treatment is initiated after symptom onset (SAR_{cur}, orange) or before symptom onset (SAR_{pep}, green). **(D)** SAR, SAR_{cur}, and SAR_{pep} values according to different values of m. **(E-F)** Generation time distribution for SAR=40% and SAR=80% respectively. **(A-B)** : Simulations conducted for $m = 4.1 \times 10^{-6}$ *mL/copies* (see Eq.(2)), corresponding to SAR= 40%.

Major comments:

▪ **The proposed multiscale model consists of a viral dynamics model and a transmission model, each parameterized using different datasets or assumptions. As a result, I find that both the model structure and the values assigned to its parameters lack complete validation.**

We have now added in Table S1 the value and reference for all parameters used, which ensures a full reproducibility of our results on both within- and between-host components, and we describe them below.

More broadly, the reviewers have commented upon the fact that the model parameters would be calibrated on SARS-CoV-2 pre-Omicron virus. In this revised manuscript we have clarified that the

purpose of our model is **not to focus on a specific variant or a specific virus**. Rather, we propose a model that is sufficiently flexible to reproduce the main patterns of viral dynamics observed for different acute respiratory viruses. If our main analysis is indeed inspired by the viral dynamics of SARS-CoV-2 pre-Omicron (with a peak viral load that coincides with symptom onset and occurs 5 days after infection), we also evaluate the results obtained with different time to peak (1-, 3-, 5- and 7-days post infection), and a shorter incubation period of 3 days. This allows us to present in the final figure of the manuscript a general analysis that can apply to different respiratory viruses (SARS-CoV-2 Omicron, IAV, RSV, HRV). Further, and this is also an essential aspect of our analysis, our analysis is not restricted to one specific value for the secondary attack rate (SAR), but rather **evaluates a large range of SAR values between 5 and 97%.** Rather than focusing on a specific virus and transmissibility here, we therefore consider **all potential levels of contagiousness**.

Another important remark was to ensure reproducibility of model simulations. Viral kinetic parameters have been previously estimated on more than 4,000 SARS-CoV-2 infected individuals ([1], full paper awaiting approval from Regeneron for submission), with data originating from two clinical studies (NCT04425629 and NCT04666441) evaluating the efficacy of the monoclonal antibody combination REGN-COV2 against SARS-CoV-2. Parameters are summarized in S1 Table and reproduce the typical pattern of viral kinetics of SARS-CoV-2 pre-Omicron virus, with a peak viral load at day 5 post infection and a median time to viral clearance of 20 days, i.e., 15 days post symptom onset (see [10] for a meta-analysis of viral kinetic profiles).

For the between-host component, the model relating the probability of transmission, p(t), during a contact between an uninfected individual and an infected individual with viral load V(t) was proposed by Ke et al [2]:

$$p(t) = 1 - e^{-MV(t)^h}$$

In order to account for the fact that the transmission is not simply proportional to viral load level but depends on many other factors, we generalized this model by assuming that M is log-normally distributed with a mean m and a standard deviation fixed to 0.49, as estimated in [3]. We found that m varying from 4×10^{-7} to 1×10^{-4}mL/copies mimic SAR ranging from 5 to 97%.

In order to clarify all these hypotheses, and following the comment from reviewer 1, we added in Table 1 a description of the main model hypotheses and we rewrote all sections alluding to model parameters (see sections "Model calibration", "Exploring a secondary Attack Rate that depends on household size", "Intervention strategies and measure of their effectiveness" and "Evaluating the impact of different viral kinetic patterns and viruses" in Method section).

・The model incorporates numerous assumptions, making it difficult for readers to discern which assumptions are reasonable or less reasonable, and how they may influence the study's conclusions.

We thank you for this important comment and apologize if model hypotheses were not clear enough. We have now proposed a new Table 1 that provides a detailed description of model hypotheses and parameters, and how these parameters were modified to explore a wide range of virus characteristics (see below).

Model component	Main model	Alternative models
**Viral dynamic model**		
Viral kinetic model	Target cell model with an innate immune response	NA
Median time to peak viral load	5 days	1, 3 and 7 days
Median time to symptom onset (Incubation)	5 days	3 days
**Transmission model**		
Probability of virus transmission during a high risk contact at time t	$p(t) = 1 - e^{M \times V(t)^{0.49}}$<br>$M \sim \log\text{-}\mathcal{N}(m, 0.85^2)$	NA
Secondary Attack Rate (SAR)	40% ($m = 4 \times 10^{-7}$mL/cp)<br>80% ($m = 1 \times 10^{-4}$ mL/cp)	5-97% ($m = 4 \times 10^{-7}$-10^{-4} mL/cp)
Household size (S)	2 to 6<br>(distribution based on French demographics)	NA
Number of high risk contacts between any two household members	2 per day	NA
Impact of household size on SAR	none	SAR decreases with S
**Antiviral treatment**		
Mechanism of action	Inhibits virus production	NA
Antiviral efficacy	99%	50% and 90%
**Treatment strategies**	**Index individual**	**Household members**
I_0 (reference)	No treatment	No treatment
I_{cur}	Within 5 days after symptom onset	No treatment
I_{pep}	Before symptom onset	No treatment
$I_{cur} + H_{pep}$	Within 5 days after symptom onset	Simultaneous initiation in all household members
$I_{pep} + H_{prep}$	Before symptom onset	Simultaneous initiation in all household members

Table 1. Main assumptions of the model

Review 2

While the overall assertions of the paper are relatively clear, there appears to be a lack of description of the methodology and original data from which their results were derived, and I recommend that the manuscript be revised to clarify the basis and accuracy of the assertions.

We thank the reviewer for the thoughtful reading of our article and for the very useful comments. They were all carefully considered and we believe this new version is much clearer and improved.

Major comments:

1. In the model with antiviral treatment placed between Lines 175 and 176, the description of I_t>tx is not provided. The definition of this variable may change the meaning of the model, so please add it to the text. And tx must be subscripted as t_x.

This has been corrected.

2. Regarding the population parameters in the S1 Table, information on which parameters were estimated from the data is missing from the text and needs to be provided. It also appears that there are no published values for the patient's viral load from the reference 20 (Conrado JD et al. 2022). If so, it seems that the data availability needs to be corrected or data should be provided according to journal guidelines. At least, the authors need to show what the data fitting results in.

We have now added in Table S1 the value and reference for all parameters used, which ensures a full reproducibility of our results on both within- and between-host components, and we describe them below.

More broadly, the reviewers have commented upon the fact that the model parameters would be calibrated on SARS-CoV-2 pre-Omicron virus. In this revised manuscript we have clarified that the purpose of our model is **not to focus on a specific variant or a specific virus**. Rather, we propose a model that is sufficiently flexible to reproduce the main patterns of viral dynamics observed for different acute respiratory viruses. If our main analysis is indeed inspired by the viral dynamics of SARS-CoV-2 pre-Omicron (with a peak viral load that coincides with symptom onset and occurs 5 days after infection), we also evaluate its predictions with different time to peak (1-, 3-, 5- and 7-days post infection), and a shorter incubation period of 3 days. This allows us to present in the final figure of the manuscript a general analysis that can apply to different respiratory viruses (SARS-CoV-2 Omicron, IAV, RSV, HRV). Further, and this is also an essential aspect of our analysis, our analysis is not restricted to one specific value for the secondary attack rate (SAR), but rather **evaluates a large**

range of SAR values between 5 and 97%. Rather than focusing on a specific virus and transmissibility here, we therefore consider **all potential levels of contagiousness**.

Another important remark was to ensure reproducibility of model simulations. Viral kinetic parameters have been previously estimated on more than 4,000 SARS-CoV-2 infected individuals ([1], full paper awaiting approval from Regeneron for submission), with data originating from two clinical studies (NCT04425629 and NCT04666441) evaluating the efficacy of the monoclonal antibody combination REGN-COV2 against SARS-CoV-2. Parameters are summarized in S1 Table and reproduce the typical pattern of viral kinetics of SARS-CoV-2 pre-Omicron virus, with a peak viral load at day 5 post infection and a median time to viral clearance of 20 days, i.e., 15 days post symptom onset (see [10] for a meta-analysis of viral kinetic profiles).

For the between-host component, the model relating the probability of transmission, p(t), during a contact between an uninfected individual and an infected individual with viral load V(t) was proposed by Ke et al [2]:

$$p(t) = 1 - e^{-MV(t)^h}$$

In order to account for the fact that the transmission is not simply proportional to viral load level but depends on many other factors, we generalized this model by assuming that M is log-normally distributed with a mean m and a standard deviation fixed to 0.49, as estimated in [3]. We found that m varying from 4×10^{-7} to 1×10^{-4}mL/copies mimic SAR ranging from 5 to 97%.

In order to clarify all these hypotheses, and following the comment from reviewer 1, we added in Table 1 a description of the main model hypotheses and we rewrote all sections alluding to model parameters (see sections “Model calibration”, “Secondary Attack Rate and household size”, “Intervention strategies and measure of their effectiveness” and “Evaluating the impact of different viral kinetic patterns” in Method section).

3. No information is provided on how to choose a value for t_x, the time to start treatment. Just the treatment strategy is specified as treatment before onset and within 5 days of symptom onset, however, how t_x is varied in this context is important information. Relatedly, the choice of incubation period tau is unclear: S1 Table says it is log-normally distributed, but looking at S6 Fig, it appears that there are no cases with incubation period shorter than 3 days, as described in the text. Does this mean that the lognormal distribution has been changed to a left-truncated distribution?

The reviewer is correct that the timing of treatment initiation is crucial. To explore the impact of the timing of treatment initiation with respect to symptom onset, different strategies were tested that we now explicitly describe in Table 1 (see last comment to reviewer 1).

We clarified the choice for the incubation period in the methods as follows: “*In the first analysis we assume a log-normal distribution for the incubation period with a median time of 5 days and a*

standard deviation of 0.125, meaning that 90% of patients have an incubation period ranging between 4 and 6 days as observed for SARS-CoV-2 pre-Omicron variants [8]. We then generalize the model with a shorter incubation period of 3 days, as observed for SARS-CoV-2 Omicron variants [8].” This, by the way, explains the observation of the reviewer that all 30 sampled individuals shown in S6 fig (now S7) had symptom onset occurring more than 3 days after infection.

4. In Figures 2B-C, P(t) and SAR are calculated for the no treatment and treatment cases (and treatment strategies), respectively, while in the subsequent results the SAR is fixed (i.e., using the value of the SAR for the no treatment case) and the effect of antiviral drugs is discussed. To avoid reader confusion, please eliminate the mention of P(t) and SAR in Figure 2 or separate the definitions of SAR for no treatment and treatment in the Methods section. Alternatively, for results after Figure 2, it should be explicitly stated in all corresponding sections that the results are for the no treatment SAR.

We thank the reviewer for this remark. We have now modified Figure 2 to distinguish the baseline SAR, in absence of treatment, and the SAR when treatment is considered.

5. Referring to the definition of AUCVL in equation (5), it appears that the variable C is the number of infected cases for each index case (each household). Under this definition, the "FAR" provided in Line 333 and Figure 3B appears to be the expected value of the FAR, so if necessary, please explicitly state "expected value of FAR" in the corresponding location. If this understanding is correct, please also add it to the definition of C on Line 219.

We thank the reviewer for raising that point. This has been corrected throughout the text.

6. Please provide the definition of "effectiveness" in Figure 5 and Figure 7. The interpretation may vary depending on whether the number of infections or AUCVL for no treatment or treatment is calculated and compared within families with the same viral dynamic’s parameters, or whether the means of the two groups are compared. In particular, I think it is also necessary to include the procedure on how the effect of prevention of infection per unit of treatment, described in Lines 388 to 392, is excluded.

The definition was indeed lacking and we thank the reviewer for raising it. We rewrote the methods and it now reads as follows (see section “Intervention strategies and measure of their effectiveness” in Methods):

Next, for each of these strategies, we simulated outbreaks in a large number of households, N_{sim}*, using the distribution of household sizes in France (see below) and this provided us with an (expected)*

number of infection $(N_{inf} = \frac{1}{N_{sim}} \times \sum_{h=1}^{N_{sim}} c_h)$, *virological burden* $(AUCVL = \frac{1}{N_{sim}} \times \sum_{h=1}^{N_{sim}} AUCVL_h)$ *and transmission rate:* $FAR_h = \frac{1}{N_{sim}} \times \sum_{h=1}^{N_{sim}} FAR_h$.

We then calculated the effectiveness of each strategy I_{tx}*, as the relative reduction in the mean outcome compared to that obtained when no treatment is given,* I_0*. Thus, an effectiveness of 50% in the transmission rate implies that a strategy reduces by half the mean FAR as compared to a strategy where no treatment is used:* $1 - \frac{FAR^{I_{tx}}}{FAR^{I_0}}$.

Per your comment we also clarified the effect of prevention of infection per unit of treatment was calculated

We also calculated for each strategy I_{tx} *the number of cases avoided per number of treatments administered as the reduction in the total number of infected individuals as compared to no treatment (*I_0*), divided by the total number treatment courses given, noted* $N_{tot} = \sum_{h=1}^{N_{sim}} n_h$*, as:*

$$\frac{1}{N_{tot}} \times \sum_{h=1}^{N_{sim}} {c_h}^{I_0} - {c_h}^{I_{tx}}$$

where n_h *is the number of treatments given in household h which can be either equal to 1 (*I_{cur} *or* I_{pep} *strategies) or* S_h*, the size of the household h (*$I_{cur} + H_{pep}$ *or* $I_{pep} + H_{prep}$ *strategies).*

In the discussion we also point out to the limitation of PEP strategies, which do not integrate the possibility that index case may not be infected

"Treating individuals in post exposure prophylaxis however increases the risk to treat uninfected individuals, and in turn increases the risk of over-treatment. While we recognize that this may lead to overestimate the cost-effectiveness of I_{pep} *strategies, we note that this could be mitigated by including only individuals with a positive PCR test, as the time between infection and first positive nasal swab PCR test is very short and is between 1 and 3 days for Influenza or SARS-CoV-2 viruses ([17], [18] and [19])".*

7. Please provide the mean and variance of the FAR and AUCVL from which the authors calculated the sample size in Figure 6, as both affect the calculation of sample size using the t-test and thus help interpret the results of the sample size changes obtained. Also, the discussion of clinical trials restricted to families with four or more family members, shown in Lines 412 to 414 and Lines 522 and 525, is not an appropriate discussion if the variances are not provided. More to this point, the sample size in the clinical trial when limited to families with four or more members needs to be shown for this discussion.

We apologize for this omission. We now better describe the calculation (see section "Designing a clinical trial to evaluate the effectiveness of an antiviral strategy" in Methods):

"We finally evaluate the number of households that would be required to demonstrate, in a clinical trial, a significant effectiveness of the different antiviral strategies, considering as primary endpoints either the mean Final Attack Rate (FAR) or the mean virological burden (AUCVL). We consider a controlled study versus placebo (no treatment), with a 1:1 randomization on households, a type I error of 5% (two-sided test) and a statistical power of 90 % for. AUCVL was treated as a continuous random variable normally distributed and a t-test was used to assess difference between treated and untreated, while FAR was analyzed as a discrete random variable using a binomial test."

We now provide the distribution of AUCVL and transmission rate, which, for the sake of graphical representation, are broken down by SAR values and household size (Figure below). We also provide the expected number of households required in the case of a large household with 4 members (S9 Fig). Per your comment we also give below the mean variance observed in the resulting AUC values for all the explored SAR values. However, we found that this figure is too technical and would not add to the manuscript and therefore was not added in the supplementary materials. We also provide the AUCVL and FAR distributions for the particular case of SAR = 40% for household sizes of 2, 4 and 6 (S10 Fig.).

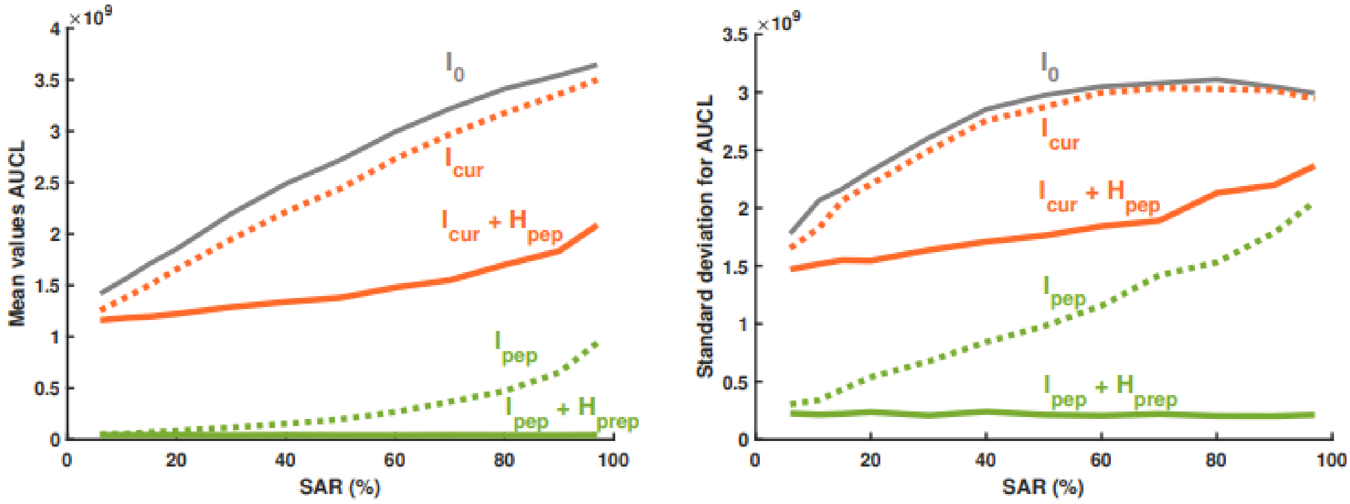

Fig. Mean value and standard deviation for AUCVL. These values were calculated for untreated households (gray) and treated households, according to a strategy where only the index is treated (dashed line), or all household members are treated (continuous line) for both scenarios- of index: treatment initiated after symptom of index (orange) and treatment initiated before symptom of index (green).

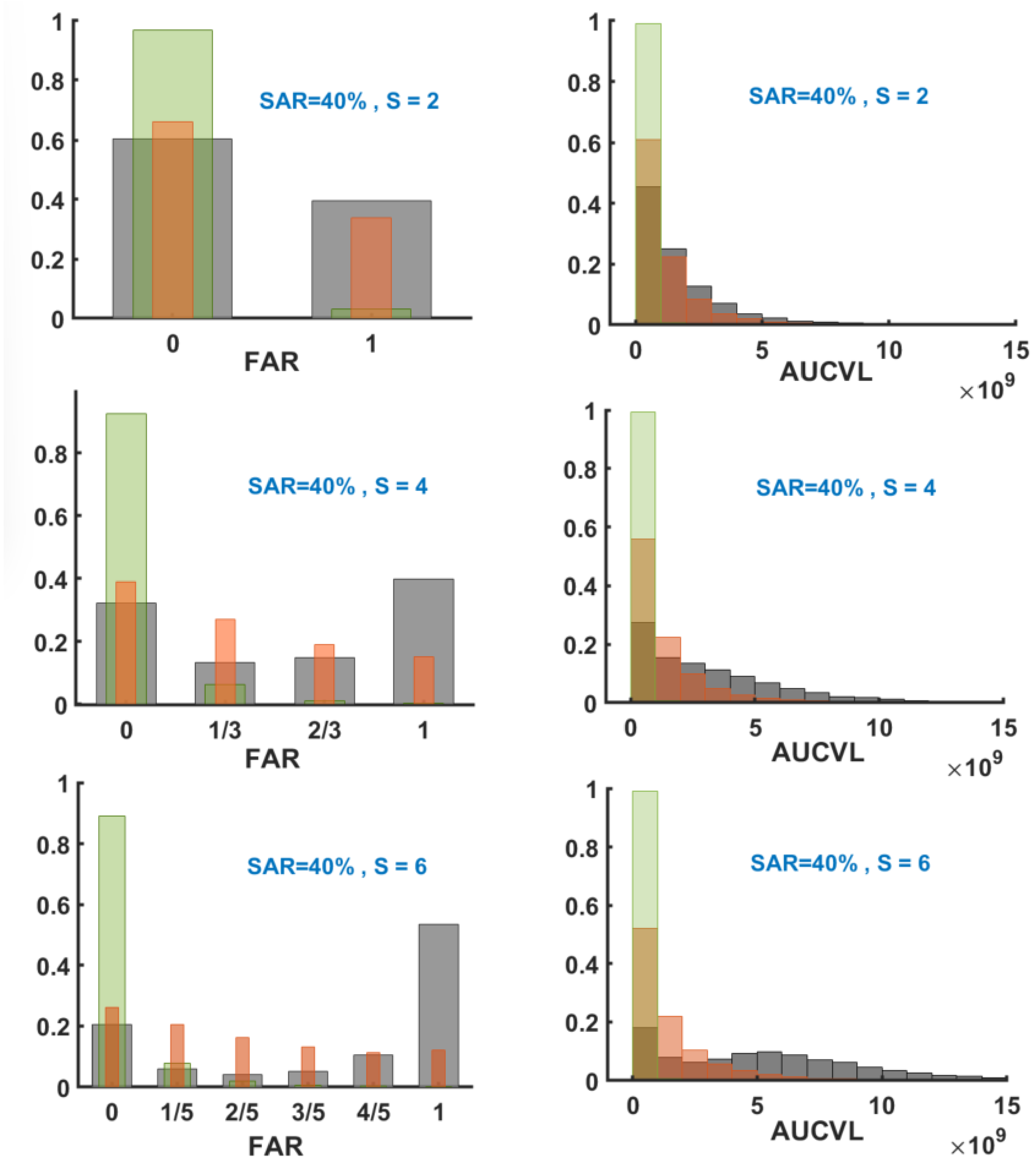

S10 Fig. AUCVL and FAR distributions for SAR= 40% and for household sizes 2, 4 and 6. Distributions for untreated households I_0 (gray) and all households treated are represented for both AUCVL (up) and FAR (bottom) with $I_{cur} + H_{pep}$ in orange and $I_{pep} + H_{prep}$ in green.

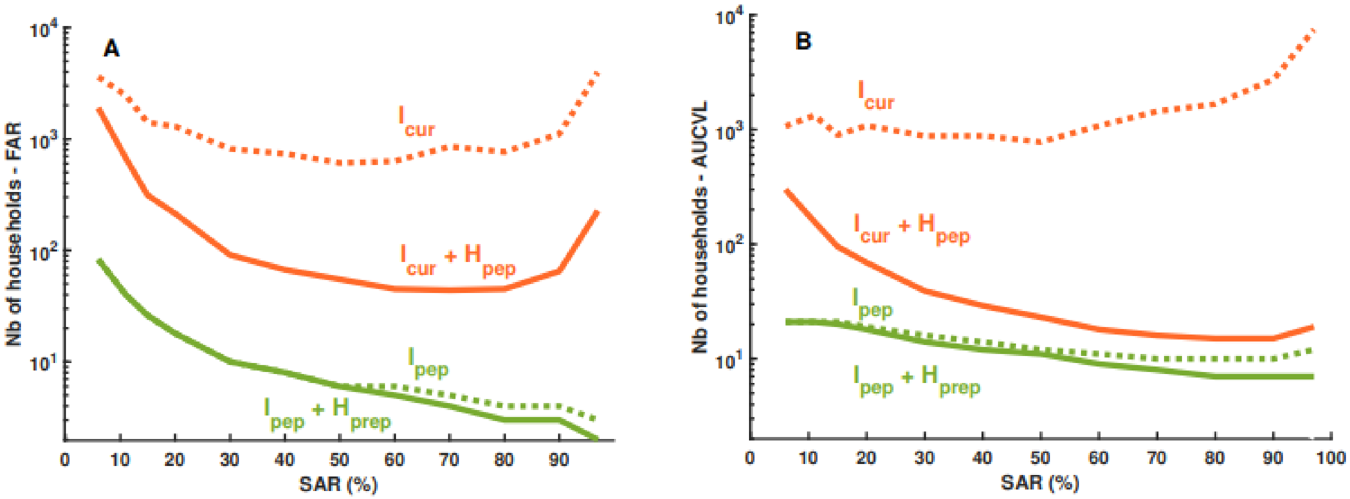

S9 Fig. **Number of households of size 4 and larger required to achieve 90% statistical power in 1:1 randomized clinical trial vs placebo (no treatment)**. (**A**) Number of households required to demonstrate a reduction in the Final Attack Rate (FAR). (**B**) Number of households required to demonstrate a reduction in the virological burden (AUCVL).

8. How is the treatment effectiveness calculated for the modified SAR shown in S4 Fig and S7 Fig? According to the definition of modified SAR given in the Methods section, this value seems to vary depending on the number of people in the household. In other words, even if one parameter m is chosen to define the distribution of M representing the impact of viral load on transmission, the SAR value will change depending on the number of people in the household. In the unmodified SAR, the SAR is uniquely determined by fixing the parameter m, against which the treatment effectiveness is calculated by the distribution of the number of people in French households. However, under a SAR that varies with the number of people in the household, it appears that treatment effectiveness cannot be calculated under a particular modified value of SAR.

We apologize if this was not clear enough and was rephrased as follows (section "Secondary Attack Rate and household size" in Methods):

Importantly, our model assumes that SAR is independent of the household size. As a large household size increases the risk of tertiary transmission (i.e., not originating from the index case), our model predicts that FAR increases with household size (Fig 3B). This may however overlook the existence of mechanisms that limit the spread of the virus in large households, such as the fact that contact rates are not homogeneous, as reported in some epidemiological studies [11]-[16].

We therefore also considered in a sensitivity analysis a model where SAR decreases with the size of the household size. Noting SAR the value observed for S=2, as before, we note SAR_S *the SAR for household size greater than 2 and we assume that* $SAR_S = \frac{SAR}{\sqrt{S-1}}$ *(S ≥ 3). Thus, in this setting, we used a different value of m in each household size, relying on the relationship between m and SAR*

that was established above. Simulations show that the expected value of FAR is much less sensitive to the household sizes (see S1 Fig.).

9. Please provide viral dynamics as shown in Figure 1 and S6 Fig for the discussion of the impact of multiple treatment effects, epsilon = 0.9 or 0.5 in lines 424 to 434.

We now provide different figures for epsilon = 90% and 50% in the Supplementary as S5 Fig.

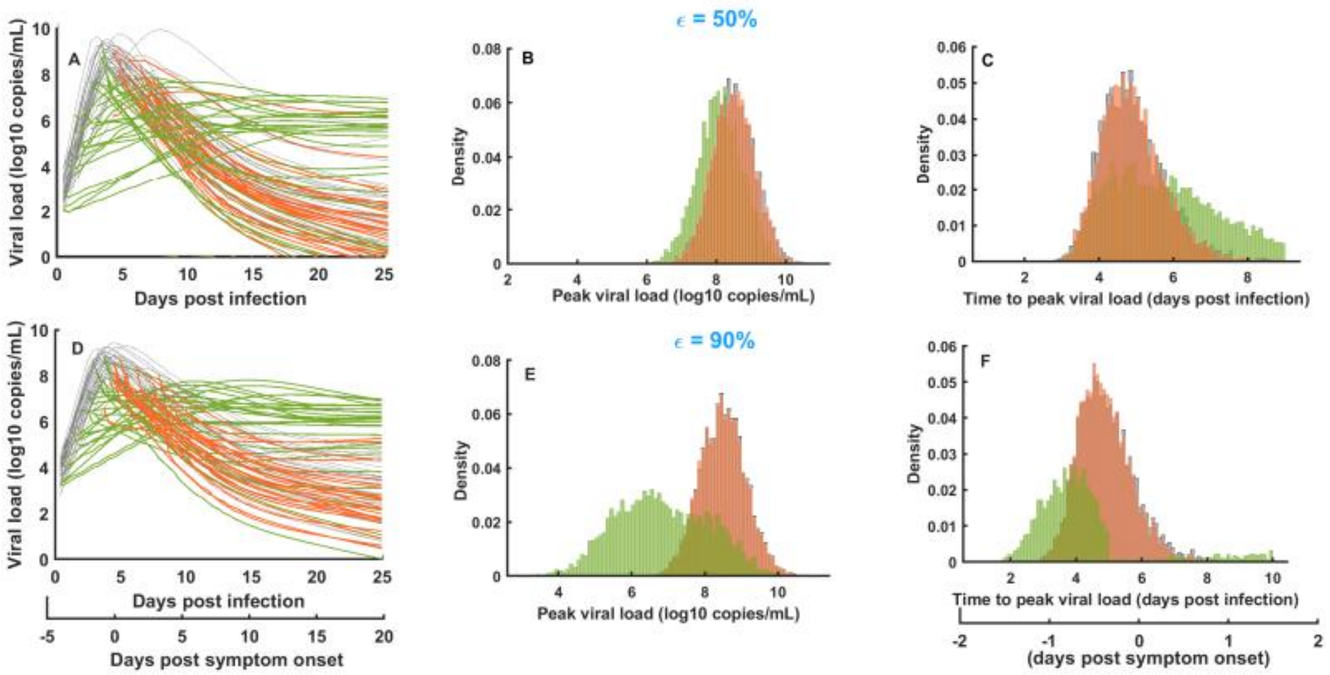

S5 Fig. Viral dynamics with lower antiviral efficacy. (**A, D**) Individual viral dynamic profiles predicted by the model in 30 individuals (Eq. (2)), that are either left untreated (I_0, gray), treated within 5 days after symptom onset (I_{cur}, orange), or treated within 5 days after infection (I_{pep}, green). All parameters are given in S1.Table. (**B, E**) Distribution of the peak viral load predicted by the model. (**C, F**) Distribution of the time to peak viral load predicted by the model. Top: $\epsilon = 50\%$; bottom: $\epsilon = 90\%$.

10. In Lines 435 – 563 and Figure 7, the delay between symptom onset and time to peak viral load is not appropriate to explain the differences in viral infection dynamics because both the incubation period and time to peak may affect effectiveness. For the sake of consistency in the discussion, I recommend showing this as the time of the peak of the viral load, as shown in Lines 294-304 and S3 Table.

We modified both figures in Fig 7. and S8 Fig.

Minor comments:

1. What does R_0 mean in S1. Fig? It seems to be different from R_0 in S1 Table, which is calculated from model parameters and not depending on SAR.

We now use a different notation to distinguish the epidemiological R0 from the within-host R0, noted R_0^{intra} .

The following comments have been taken into account in the paper

2. The simultaneous equations between lines 175 and 176 are not numbered in the equation, but the reference elsewhere is to Eq. (5), which should be corrected.

3. In Line 182 and 184 and S1 Table, epsilon is shown as a percentage value, but in the mathematical model definition, it appears to be between 0 and 1. If this is correct, I recommend that it be stated accurately, i.e. epsilon = 0.99, 0.9 or 0.5.

4. In Line 192, please revise "see (2)" to "see Eq. (2)".

5. In Line 196, please delete the duplicate "that".

6. Please add an explanation of the meaning of "gray" for Figure 1BC. The presence of the gray distribution may be missed because it overlaps with other colors.

The gray distributions are under the orange one as the treatment is mostly initiated after the peak load time.

7. It would be helpful to refer to the panel of figures to aid the reader's understanding. In Line 363, "S3 Fig" can be "S3A and B Fig", in Line 373, 380 "S3 Fig" can be "S3D and E Fig", and in Line 407, "Fig. 6" can be "Fig. 6B". Please check the journal's guidelines for reference style of figures.

8. Please specify that beta_5d, delta_5d, p_5d in S3 Table is the same as beta, delta, p in S1 Table.

We added the explanation in the table of the supplementary.

9. In my environment, there was a garbled character in line 386. Looks like "S¿4", this might be "S > 4".

10. In Lines 405-407, please clarify that this sentence is for treatment initiated after symptom of index.

11. In Line 437, referring Figure 1 for cases with peak viral load at 5 days.

12. In S6 Fig please provide the difference of colors as provided in Figure 1

13. In Lines 445 – 448, Lines 450 – 454, are the percentage values (25-50% and 50-75%) shown here SAR?

These values are for the effectiveness (explanation added in the main paper in these lines).

References

[1] Conrado JD, Patel K, Lin KJ, Wang Y, Bhongsatiern J, Kamal MA et al. Viral kinetics in COVID-19 outpatients treated with CASIRIVIMAB+IMDEVIMAB combination. Conference on Retroviruses and Opportunistic Infections, 2022.

[2] Ke R, Zitzmann C, Ho DD, Ribeiro RM and Perelson AS. In vivo kinetics of SARS-CoV-2 infection and its relationship with a person's infectiousness. Proceedings of the National Academy of Sciences, 2021, 118(49), e2111477118.

[3] Marc A, Kerioui M, Blanquart F, Bertrand J, Mitj`a O, Corbacho-Monn´e M et al. Quantifying the relationship between SARS-CoV-2 viral load and infectiousness. Elife, 2021, 10, e69302.

[4] Hammond J, Leister-Tebbe H, Gardner A, Abreu P, Bao W, Wisemandle W et al. Oral nirmatrelvir for high-risk, nonhospitalized adults with Covid-19. New England Journal of Medicine, 2022, 386(15), 1397-1408.

[5] Weinreich DM, Sivapalasingam S, Norton T et al. REGEN-COV antibody combination and outcomes in outpatients with Covid-19. New England Journal of Medicine, 2021, 385(23), e81

[6] Ali ST, Wang L, Lau EHY et al. Serial interval of SARS-CoV-2 was shortened over time by nonpharmaceutical interventions. Science, 2020, 369(6507) : 1106-1109.

[7] Manica M, De Bellis A, Guzzetta G et al. Intrinsic generation time of the SARS-CoV-2 Omicron variant: An observational study of household transmission. Lancet Reg Health Eur, 2022, 19:100446.

[8] Wu Y, Kang L, Guo Z, Liu J, Liu M and Liang W. Incubation period of COVID-19 caused by unique SARS-CoV-2 strains: a systematic review and meta-analysis. JAMA network open, 2022, 5(8), e2228008-e2228008.

[9] Hart WS, Abbott S, Endo A, et al. Inference of the SARS-CoV-2 generation time using UK household data. Elife. 2022, 11: e70767. doi: 10.7554/eLife.70767.

[10] Cevik M, Tate M, Lloyd O, Maraolo AE, Schafers J and Ho A. SARS-CoV-2, SARS-CoV, and MERS-CoV viral load dynamics, duration of viral shedding, and infectiousness: a systematic review and meta-analysis. The lancet microbe, 2021, 2(1), e13-e22.

[11] Li F, Li YY, Liu MJ, Fang LQ, Dean NE, Wong GW et al. Household transmission of SARS-CoV-2 and risk factors for susceptibility and infectivity in Wuhan: a retrospective observational study. The Lancet Infectious Diseases, 2021, 21(5), 617-628.

[12] Bernal JL, Panagiotopoulos N, Byers C, Vilaplana TG, Boddington N, Zhang XS et al. Transmission dynamics of COVID-19 in household and community settings in the United Kingdom. MedRxiv, 2020

[13] Arnedo-Pena A, Sabater-Vidal S, Meseguer-Ferrer N, Pac-Sa MR, Mañes-Flor P, Gascó-Laborda JC et al. COVID-19 secondary attack rate and risk factors in household contacts in Castellon (Spain): Preliminary report. Enfermedades Emergentes, 2020 19(2), 64-70.

[14] Kuwelker K, Zhou F, Blomberg B, Lartey S, Brokstad KA, Trieu MC et al. Attack rates amongst household members of outpatients with confirmed COVID-19 in Bergen, Norway: a case-ascertained study. The Lancet Regional Health-Europe, 2021, 3, 100014.

[15] Jing QL, Liu MJ, Zhang ZB, Fang LQ, Yuan J, Zhang AR et al. Household Secondary Attack Rate of COVID-19 and Associated Determinants. The Lancet Infectious Diseases. 2020; 20(10): 1141-1150.

[16] Tibebu S, Brown KA, Daneman N, Paul LA and Buchan SA. Household secondary attack rate of COVID-19 by household size and index case characteristics. MedRxiv, 2021.

[17] Baccam P, Beauchemin C, Macken CA, Hayden FG and Perelson AS.Kinetics of influenza A virus infection in humans. Journal of virology, 2006, 80(15), 7590-7599.

[18] Carrat F, Vergu E, Ferguson NM, Lemaitre M, Cauchemez S, Leach S and Valleron AJ. Time lines of infection and disease in human influenza: a review of volunteer challenge studies. American journal of epidemiology, 2008, 167(7), 775-785.

[19] Killingley B, Mann AJ, Kalinova M, Boyers AI, Goonawardane N et al. Safety, tolerability and viral kinetics during SARS-CoV-2 human challenge in young adults. Nature Medicine, 2022, 28(5), 1031-1041.

[20] Madewell ZJ, Yang Y, Longini IM, Halloran ME and Dean NE. Household Transmission of SARS-CoV-2: A Systematic Review and Meta-analysis. JAMA network open. 2020 ;3(12): e2031756-e2031756.

[21] Jing QL, Liu MJ, Zhang ZB, Fang LQ, Yuan J, Zhang AR et al. Household Secondary Attack Rate of COVID-19 and Associated Determinants. The Lancet Infectious Diseases. 2020; 20(10): 1141-1150.

[22] Layan M, Gilboa M, Gonen T, Goldenfeld M, Meltzer L et al. Impact of BNT162b2 vaccination and isolation on SARS-CoV-2 transmission in Israeli households: an observational study. American journal of epidemiology, 2022, 191(7), 1224-1234.

[23] López-Muñoz I, Torrella A, Pérez-Quílez O, Castillo-Zuza A, Martró E et al. SARS-CoV-2 Secondary Attack Rates in Vaccinated and Unvaccinated Household Contacts during Replacement of Delta with Omicron Variant, Spain. Emerging infectious diseases, 2022, 28(10), 1999-2008.

[24] Chen D, Lau YC, Xu XK, Wang L et al. Inferring time-varying generation time, serial interval, and incubation period distributions for COVID-19. Nature communications, 2022, 13(1), 7727.

[25] Hart WS, Miller E, Andrews NJ et al. Generation time of the alpha and delta SARS-CoV-2 variants: an epidemiological analysis. The Lancet Infectious Diseases, 2022, 22(5), 603-610.
