# Supplementary material for: Modelling the effectiveness of antiviral treatment strategies to prevent household transmission of acute respiratory viruses
Source: PLoS Comput Biol. 2024 Dec 5;20(12):e1012573. doi: 10.1371/journal.pcbi.1012573 (PMC11620401; doi:10.1371/journal.pcbi.1012573)
Supplement: Attachment — Submitted filename: Response to Reviewers 2.pdf [file pcbi.1012573.s018.pdf]

Modelling the effectiveness of antiviral treatment strategies to prevent household transmission of acute respiratory viruses

Hind Zaaraoui[1,*]***, Xavier Duval***[1]***, Bruno Hoen***[2]***, Lulla Opatowski***[3,+]***, Jérémie Guedj***[1,+]

1 Université Paris Cité, IAME, INSERM, F-75018 Paris, France.
2 Université de Lorraine, 54000 Nancy, France.
3 Université de Versailles Saint Quentin, Institut Pasteur, Inserm, Paris, France.

***** hind.zaaraoui@inserm.fr
+These authors also contributed equally to this work.

Responses to referees and editor

We thank the reviewers and the reviewing editor for their comments.

Please find our answers below each comment.;

Editor

1. The Authors have improved the analyses, provided supporting results concerning the validity of the connection between viral dynamics and transmission, and clarified the model assumptions. The Authors are expected to address all further criticisms by Reviewer #1. In particular, please clarify the start time of treatment and $C_{[0,t]}$:

- We apologize for this lack of clarity, we now clarify these aspects in the section "Intervention strategies and measure of their effectiveness" and in Table 1:

"We consider four treatment strategies. In the first scenario, noted I_{cur}, the index individual initiates treatment within 5 days from symptom onset, assuming a uniform distribution for treatment initiation between 0 and 5 days after symptom onset. The second scenario, noted I_{pep}, assumes that treatment is administered before symptom onset as a post-exposure prophylaxis, assuming again a uniform distribution for treatment initiation between infection time ($t=0$) and symptom onset. Then we consider the same treatment strategies, assuming now that treatment is given not only to the index individual, but also and simultaneously to all household members, regardless of their infection status, as either a post-exposure prophylaxis (when the index is already symptomatic, noted $I_{cur} + H_{pep}$), or as a pre-exposure prophylaxis (when the index is not yet symptomatic, noted $I_{pep} + H_{prep}$) (Table 1)."

Treatment strategies	Index individual	Household members
I_0 (reference)	No treatment	No treatment
I_{cur}	Treatment randomly initiated within 5 days from symptom onset	No treatment
I_{pep}	Treatment randomly initiated before symptom onset	No treatment
$I_{cur} + H_{pep}$	Treatment randomly initiated within 5 days from symptom onset	Simultaneous initiation in all household members
$I_{pep} + H_{prep}$	Treatment randomly initiated before symptom onset	Simultaneous initiation in all household members

Table 1. Main assumptions of the model

- We have clarified the definition of the probability of contact to avoid multiple notations and we no longer introduce $\boldsymbol{C_{[0,t]}}$. The section "Transmission model" now reads:

"As household contacts are not unique and are repeated, we note $P(\infty) = lim_{\{t\to\infty\}} P(t)$ the probability that at least one of these contacts leads to an infection, given by: $P(t) = 1 - \prod_{u\in[0,t]}(1 - \mathrm{p(u)})$, and we assume without loss of generality 1 contact every 12 hours (i.e., 2 contacts per day)."

Review 1

1. In the 3rd to 6th lines in Abstract, "During an outbreak, more aggressive strategies involving pre- or post-exposure prophylaxis (PEP or PrEP) could be employed to further reduce the risk of severe disease but also prevent transmission to household contacts.", "PEP" and "PrEP" may be opposite.

We apologize for this typo, it now reads: "During an outbreak, more aggressive strategies involving pre- or post-exposure prophylaxis (PrEP or PEP) could be employed to further reduce the risk of severe disease but also prevent transmission to household contacts."

2. I think it is better to mention as a limitation that the transmission model (p(t)) formula itself is not a complete generalization of respiratory infectious diseases, since it is estimated from COVID-19 data and has not been validated in other viral infections. Even among respiratory infections, the importance of the types of droplets and their symptoms may differ among different types of infections.

We thank you for this important comment. We added in the Discussion section:

“First, a power law model was used to relate the viral load at time *t* and the risk of transmission (see Eq. 2). Although this model was successfully used to reproduce the risk of transmission in SARS-CoV-2 [1], and that we here explored a large range of parameter values to encompass different levels of virus transmissibility, this model may not universally apply to all ARI. Such differences could for instance reflect the importance of the types of droplets causing the infection and/or different symptom patterns leading to different functional forms between viral load and transmission.”

3. P(∞) of the SAR should be calculated at a time large enough to be approximated as P(t_∞) in practice, so please add it to the Transmission model in Methods. It seems that C_[0,t], which is required for the calculation of SAR, is not provided.

We have clarified the definition of the probability of contact to avoid multiple notations and we no longer introduce $\boldsymbol{C}_{[\boldsymbol{0},\boldsymbol{t}]}$. The section “Transmission model” now reads:

“As household contacts are not unique and are repeated, we note $P(\infty) = lim_{\{t\to\infty\}}\, P(t)$ the probability that at least one of these contacts leads to an infection, given by: $P(t) = 1 - \prod_{u\in[0,t]}(1 - \mathrm{p(u)})$, and we assume without loss of generality 1 contact every 12 hours (i.e., 2 contacts per day).”

I could not find detailed information on how the timing of individual treatments is chosen for t_x, the start time of treatment. Table 1 only provides information on whether it is before or after symptom onset. t_x may be a constant, or it may be randomly sampled from some distribution, either way, the authors need to specify how the actual values used were determined. This needs to be provided for the simulations in Fig. 1 and Fig. 2 and for the case of household infection after Fig. 4, if different from each other.

- We apologize for this lack of clarity omission, we now added detailed information to the section “Intervention strategies and measure of their effectiveness” and in Table 1:

  “We consider four treatment strategies. In the first scenario, noted I_{cur}, the index individual initiates treatment within 5 days from symptom onset, assuming a uniform distribution for treatment initiation between 0 and 5 days after symptom onset. The second scenario, noted I_{pep}, assumes that treatment is administered before symptom onset as a post-exposure prophylaxis, assuming again a uniform distribution for treatment initiation between infection time (*t=0*) and symptom onset. Then we consider the same treatment strategies, assuming now that treatment is given not only to the index individual, but also and simultaneously to all household members, regardless of their infection status, as either a post-exposure prophylaxis (when the index is already symptomatic, noted $I_{cur} + H_{pep}$), or as a pre-exposure prophylaxis (when the index is not yet symptomatic, noted $I_{pep} + H_{prep}$) (Table 1).”

Treatment strategies	Index individual	Household members
I_0 (reference)	No treatment	No treatment
I_{cur}	Treatment randomly initiated within 5 days from symptom onset	No treatment
I_{pep}	Treatment randomly initiated before symptom onset	No treatment
$I_{cur} + H_{pep}$	Treatment randomly initiated within 5 days from symptom onset	Simultaneous initiation in all household members
$I_{pep} + H_{prep}$	Treatment randomly initiated before symptom onset	Simultaneous initiation in all household members

Table 1. Main assumptions of the model

4. Fig. S10 may be the distribution of FAR_h and AUCVL_h. The sample size discussion does not seem to be significantly affected by the variance of AUCVL_h as shown in the Letter. However, the sample size change in relation to SAR is not only determined by the AUCVL change, so I think it should be included in the supplementary information. I also think that the values of AUCVL and the standard deviation of AUCVL_h should be shown for the case restricted to households with 4 or more members, which you added to S9 Fig.

We modified the name of the distributions in the S10 Fig. to" FAR_h" and "AUCVL_h" instead of "FAR" and "AUCVL".

Per your comment, we also added a supplementary figure representing mean and standard deviation of AUCVL_h for A/ all households and B/ households of sizes of 4 and larger (S11 Fig.):

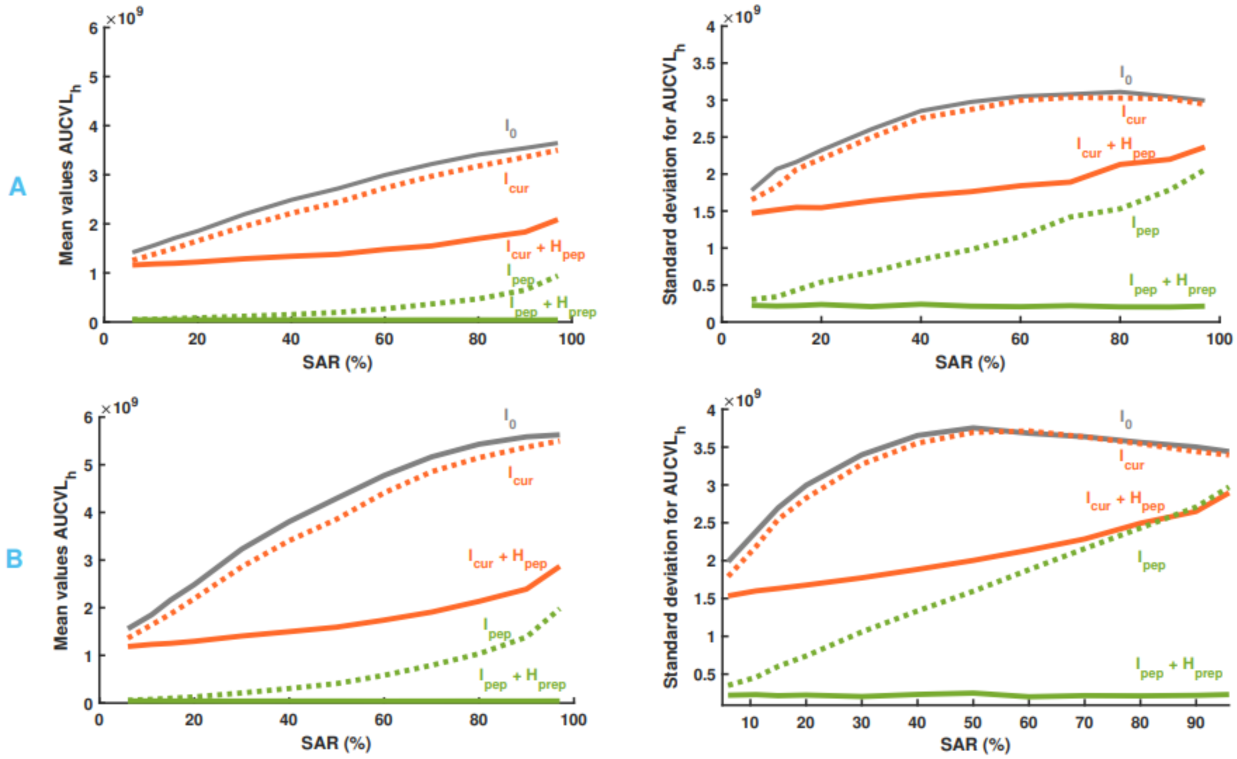

S11 Fig. Mean value and standard deviation for AUCVL_h. These values were calculated for all household (A, upper figures) and for households of size 4 and larger (B, bottom figures). Orange dashed line: I_{cur}; Orange continuous line: $I_{cur} + H_{pep}$; Green dashed line: I_{pep}; Green continuous line: $I_{pep} + H_{prep}$.

References

[1] Ke R, Zitzmann C, Ho DD, Ribeiro RM and Perelson AS. In vivo kinetics of SARS-CoV-2 infection and its relationship with a person's infectiousness. Proceedings of the National Academy of Sciences, 2021, 118(49), e2111477118.
