# Supplementary material for: Modelling the effectiveness of antiviral treatment strategies to prevent household transmission of acute respiratory viruses
Source: PLoS Comput Biol. 2024 Dec 5;20(12):e1012573. doi: 10.1371/journal.pcbi.1012573 (PMC11620401; doi:10.1371/journal.pcbi.1012573)
Supplement: Attachment — Submitted filename: cover letter for reviews jg 4.pdf [file pcbi.1012573.s019.pdf]

Jérémie Guedj
Research Director
INSERM UMR U1137,
16 rue Henri Huchard, 75018 Paris, France
jeremie.guedj@inserm.fr

Paris, September 24th 2024

Dear Editor,

Please find attached our revised manuscript entitled "Modelling the effectiveness of antiviral treatment strategies to prevent household transmission of acute respiratory viruses".

We thank you for the stimulating discussion following our last submission. Per your request, we have now entirely updated our model and results to rely only on publicly available data. We now use one the most detailed dataset of SARS-CoV-2 viral kinetic data published so far [1] and documents viral kinetics in 1443 SARS-CoV-2 infections [2]. We have added as a supplementary material all details concerning the fitting procedure and results obtained. This analysis was performed by Clarisse Schumer, who has now been added as a coauthor. All other co-authors have approved the change in authorship.

This analysis also includes both individuals infected with pre-Omicron or Omicron variants and is therefore not specific to pre-Omicron variants, as was the case in our initial submission. Per your comment, we have also clarified in the discussion that our approach is general and does not aim to discuss one specific variant. It now reads

"*First, while our central scenario relied on typical parameters of SARS-CoV-2 viral dynamics, our approach aims to provide a general understanding of the factors governing treatment efficacy against ARI and is not tailored for one specific virus. Indeed, we explored a variety of viruses, as illustrated by the large range of SAR values covering different possible ranges of virus transmissibility, and the different times to peak viral load, close to other ARI such as Influenza or RSV. Although vaccination or new variants can affect the within-host viral*

dynamics, this question was not in the scope of this study but the model can easily be adapted to any host-pathogen characteristics by changing within-host parameters.”

We hope that these changes address the last comments that remained on our manuscript and we hope that the paper is now suitable for publication in *PLoS Computational Biology*.

Best regards

Jérémie Guedj, PhD

REFERENCES

[1] Owens K, Esmaeili-Wellman S, Schiffer JT. Heterogeneous SARS-CoV-2 kinetics due to variable timing and intensity of immune responses. JCI Insight, 2024;9(9): e176286.

[2] https://github.com/lacyk3/SARS-CoV-2Kinetics
